# Autistic phenotypes and genetic testing: state-of-the-art for the clinical geneticist

**DOI:** 10.1136/jmg.2008.060871

**Published:** 2008-12-18

**Authors:** C Lintas, A M Persico

**Affiliations:** Laboratory of Molecular Psychiatry & Neurogenetics, University Campus Bio-Medico, Rome, Italy and Department of Experimental Neurosciences, IRCCS. “Fondazione Santa Lucia”, Rome, Italy

## Abstract

Autism spectrum disorders represent a group of developmental disorders with strong genetic underpinnings. Several cytogenetic abnormalities or de novo mutations able to cause autism have recently been uncovered. In this study, the literature was reviewed to highlight genotype–phenotype correlations between causal gene mutations or cytogenetic abnormalities and behavioural or morphological phenotypes. Based on this information, a set of practical guidelines is proposed to help clinical geneticists pursue targeted genetic testing for patients with autism whose clinical phenotype is suggestive of a specific genetic or genomic aetiology.

Autism spectrum disorders (ASDs) represent a heterogeneous group of neurodevelopmental disorders characterised by social and communication deficits, accompanied by repetititive and stereotyped behaviours, with onset before 3 years of age.^1^ ^2^ From a diagnostic standpoint, ASDs coincide with the *Diagnostic and statistical* manual *of mental disorders*, 4th edition (DSM-IV) criteria for pervasive developmental disorders, including autistic disorder, Asperger syndrome, childhood disintegrative disorder, Rett syndrome and “pervasive developmental disorder not otherwise specified” (PDD-NOS).^1^ ^2^ However, the concept of ASD is somewhat complementary to DSM-IV categorical diagnoses, because it underlines that autistic-like traits and behaviours represent a continual spectrum rather than clinically defined diagnostic categories.^3^ The male:female sex ratio for autistic disorder is 4:1, implying an involvement of the X chromosome and/or imprinting mechanisms. Autism is associated with seizures and mental retardation (MR) in up to 30% and 80% of cases, respectively.^4^ ^5^ A prenatal origin of the disease is supported by neuroanatomical and neuroimaging studies, showing abnormalities stemming from disturbed neurodevelopmental processes physiologically occurring during the first and second trimester of pregnancy.^6^ ^7^

Genetics strongly contributes to the pathogenesis underlying ASDs.^8^ ^9^ Rett syndrome, a monogenic disorder affecting female carriers of mutations in the *MeCP2* gene,^10^ has been described in detail previously.^11^ In this review, we focus on the remaining ASDs and especially on autistic disorder, the neuropsychiatric disorder with the highest monozygotic twin concordance rate (73–95%), the highest heritability (>90%, as estimated by twin studies) and a noticeable sibling recurrence risk (5–6% for non-syndromic autism).^8^ ^9^ In addition the presence of mild autistic traits in first-degree relatives of patients with autism points towards a strong genetic component in ASD.^3^ Linkage and association studies have identified numerous susceptibility genes, located on various chromosomes, especially 2q, 7q, 15q and the X chromosome.^8^ ^9^ The clinical heterogeneity of ASD probably reflects the complexity of its genetic underpinnings, involving multiple contributing loci, genetic heterogeneity, epistasis and gene–environment interactions.^9^ Interestingly, epidemiological studies reported an incidence of 2–5 in 10 000 newborn babies before 1985, whereas studies performed after the year 2000 converge upon rates of ASD as high as 20–60 in 10 000.^12^ ^13^ This increase may stem from the use of broader diagnostic criteria and increased attention by the medical community,^12^ ^13^ but a real increase in incidence of ASD due to environmental and epigenetic factors acting upon a genetically vulnerable background is also likely.^9^

Genetic screens represent a powerful tool when dealing with monogenic mendelian disorders, characterised by direct genotype–phenotype correlations. In the case of complex disorders, such as ASD, widespread genetic testing would not only be expensive and time-consuming, but also generally inappropriate due to the aetiological complexity described above.^9^ Nonetheless, there are at least two instances where genetic testing can be successfully used in complex disorder: to evaluate the degree of genetic susceptibility to a certain disease and to identify rare monogenic or cytogenetic forms of the disease. The appropriate use of genetic testing in these two instances is good clinical practice for the following reasons: (1) the identification of susceptibility variants can allow the implementation of prevention programmes, as exemplified by familial breast and ovarian cancers involving *BRCA1* and *BRCA2* mutations,^14^ or familial colon cancer;^15^ (2) the identification of the exact genetic cause of an otherwise unexplained disease, especially when it affects children, can significantly reduce the levels of guilt and anxiety in parents and improve their compliance with medical interventions and to rehabilitation programmes.

During the past few years, genetic research in ASDs has begun achieving some important successes, including the identification of several vulnerability loci and of a few cytogenetic abnormalities or single-base mutations able to cause autism (see below). In order to transfer this recently acquired knowledge into clinical practice, it is critical to define a set of phenotypic inclusion criteria that must be met by affected probands to justify their enrolment in a specific genetic testing programme. Accordingly, these genetic screening programmes must be designed to use clearly targeted, feasible and cost-effective strategies. We reviewed the relevant literature to look for specific correlations between ASD-causing gene mutations or cytogenetic abnormalities and clinical ASD phenotypes (mainly behavioural and/or morphological ASD phenotypes). We hope this information will be useful to guide clinical geneticists in establishing and implementing effective genetic screening programmes for those patients with ASD whose phenotype is suggestive of a specific genetic or genomic aetiology.

## SYNDROMIC AUTISM

Syndromic autism (ASD associated with a known cause) represents approximately 10% of all ASD cases and is often associated with malformations and/or dysmorphic features.^8^ ^16^ Unlike “idiopathic” or “primary” ASD, syndromic autism shows an equal male:female sex ratio.^17^ Syndromic autism can be associated with well-known genetic or genomic disorders (including fragile X syndrome, neurofibromatosis, tuberous sclerosis, untreated phenylketonuria, and Angelman, Cornelia de Lange and Down syndromes), with de novo chromosomal rearrangements detectable by karyotyping (duplication of the maternal 15q11-13 region, deletions of chromosome 2q37, 7q31, 22q11 and microdeletions of chromosome 22q11.2) and with rare environmental events (prenatal central nervous system infection by rubella or cytomegalovirus, prenatal exposure to valproic acid or thalidomide).^8^ ^16^

The recent advent of array-based high-resolution genomic analysis has dramatically increased the power to detect small de novo genomic deletions and duplications, down to a few kilobases in size, well below the threshold of detection by standard chromosomal banding and karyotyping techniques. At least four independent genome-wide studies have identified de novo microdeletions, microduplications or copy number variants (CNVs) associated with autism using array-comparative genome hybridisation (CGH) techniques using either bacterial artificial chromosome (BAC) or single-nucleotide polymorphism arrays.^18^^–^21 Jacquemont *et al*^18^ found that 8 of 29 (27.5%) patients with autism carrying microdeletions or microduplications of 1.4–16.0 Mb in size, including six de novo chromosomal rearrangements presented with dysmorphic facial features. Sebat *et al*^19^ found de novo CNVs in 10% of trios with one affected child (12 of 118) and in 1% of normal trios (2 of 196). Although the clinical characteristics of de novo CNV carriers were not described in detail, they were interestingly not found to be at increased risk of MR and some were apparently diagnosed with Asperger syndrome, suggesting that CNVs may also be associated with milder forms of autism, perhaps not accompanied by dysmorphic features. Marshall *et al*^20^ assessed 427 unrelated patients with ASD and 500 controls, finding 27 (6.3%) cases carrying de novo CNVs, which may be especially present in simplex (4/56 = 7.1%) rather than in multiplex (1/49 = 2.0%) families. Many, but not all patients, display dysmorphic features and/or neurological impairment. Christian *et al*^21^ reported the presence of 7 de novo and 44 inherited CNVs in 397 patients with ASD. The clinical features were not discussed in detail, but no evidence of signs or symptoms shared by patients carrying de novo chromosomal microdeletions or microduplications was reported. Using array-based technologies, others have reported novel and possibly recurrent microdeletion syndromes, affecting regions such as 16p11.2 and 2p15–16.1.^22^^–^24 These studies have confirmed that CNVs can be associated with a variety of clinical features, including non-ASD behavioural disorders in siblings of patients with autism. Therefore, the presence of malformations, dysmorphic features and/or severe neurological symptoms strongly points toward a possible cytogenetic origin for ASDs, but their absence does not exclude it. The progressive transfer of array-based approaches from the laboratory into clinical practice will enhance our ability to detect an increasing number of submicroscopic de novo chromosomal abnormalities.

## GENES INVOLVED IN MONOGENIC FORMS OF AUTISM

### Neuroligin 3/4 genes (*NLGN3*, *NLGN4*)

Neuroligins are cell-adhesion molecules localised postsynatptically in both glutamatergic (NLGN1, NLGN3, NLGN4, NLGN4Y) and gamma-aminobutyric acid-ergic (NLGN2) synapses.^9^ ^25^ They interact with presynaptic β-neurexin, triggering the formation of functional presynaptic structures in contacting axons. They also associate with postsynaptic scaffolding proteins, such as SHANK3, also involved in ASD (see below). The *NLGN3* and *NLGN4* genes, located at human chromosome loci Xq13 and Xq22.33, respectively, have been found to host rare mutations explaining <1% of ASD cases (table 1). Jamain *et al*^26^ reported a frameshift (D396X in NLGN4) and a missense (R451C in NLGN3) mutation in two unrelated Swedish families, both mutations inherited from unaffected mothers. In vivo studies using D396X or R451C mouse mutants showed increased inhibitory synaptic transmission and impaired social interaction,^27^ paralleled by functional studies showing defective vesicle-trafficking and synapse-induction properties.^28^^–^30 Extensive genetic screens conducted by several groups have confirmed the low frequency of neuroligin mutations among patients with ASD (7/861; 0.8%; table 1). Laumonnier *et al*^31^ found a frameshift mutation (D429X in NLGN4) in 13 affected male members of a single pedigree (counted as one instance in table 1, because they are related individuals coming from a single pedigree). Lawson-Yuen *et al*^32^ identified a deletion of exons 4–6 of *NLGN4* in an boy with autism and in his brother with Tourette syndrome; the mutation was inherited by the mother who also had neuropsychiatric disorders. Yan *et al*^33^ reported four other *NLGN4* missense mutations in patients with ASD (G99S, K378R, V403M and R704C). However, V403M and R704C were also found in unaffected siblings, suggesting they could represent rare non-synonymous variants rather than true mutations. None of 553 patients with ASD assessed in five other studies were found to carry NLG3 or NLG4 non-synonymous variants.^34^^–^38 In contrast, novel splice variants were identified in autistic individuals by Talebizadeh *et al*.^39^

**Table 1:** Neuroligin 3/4 genes and autistic spectrum disorders

Reference	Patients with ASD, n (sex)	Hemizygous mutations, n (%)	Mutations/deletions	Clinical phenotype
Jamain *et al*^26^	158 (140M,18F)	2/158 (1.26)	NLGN4: D396X,* NLGN3: R451C*	Autism or Asperger syndrome
Blasi *et al*^37^	124 (all M)	—	—	—
Vincent *et al*^34^	196 (149M,47F)	—	—	—
Laumonnier *et al*^31^	1 family with 13 affected males	13†	NLGN4: D429X*	Mental retardation, autism, PDD-NOS
Ylisaukko-oja *et al*^36^	30 (26M, 4F)	—	—	—
Yan *et al*^33^	148 (122M, 26F)	3/148 (2)	NLGN4: G99S,‡ K378R,* 403M,* R704C*	Mild to severe autism, PDD-NOS
Gauthier *et al*^35^	96 (83M,13F)	—	—	—
Wermter *et al*^38^	107 (102M, 5F)	—	—	—
Lawson-Yuen *et al*^32^	1 family (1 affected male)	1	Del NLGN4, exons 4,5,6§	Autism with motor tics
Total	861	7/861 (0.8)	—	—

ASD, autistic spectrum disorders; PDD-NOS, pervasive developmental disorder not otherwise specified.

*Mutations also found in heterozygous asymptomatic mothers and/or other unaffected family members.

†Counted as a single mutation, because these were consanguineous individuals from the same large pedigree.

‡Mutation found to be heterozygous in a female patient and in her mother (affected by a learning disability).

§Mutation found to be heterozygous in the patient’s mother (learning disorder, anxiety and depression) and his brother (Tourette syndrome).

The clinical phenotype of human NLG mutation carriers is very heterogeneous. In both pedigrees carrying the D396X and R451C mutations, two affected brothers carrying the mutated gene were diagnosed, one with Asperger syndrome (the “speech-preserved” variant of autism) and the other with autistic disorder. The extended pedigree carrying the D429X frameshift mutation in NLGN4 encompasses 10 male carriers affected with non-specific X-linked MR, two male carriers with autism and one with PDD-NOS (the autism variant not satisfying all diagnostic criteria). Individuals carrying microdeletions involving the NLGN4 may even satisfy diagnostic criteria for neuropsychiatric disorders apparently unrelated to ASDs.^32^ In addition, the G99S and K378R mutations yield broad clinical heterogeneity, ranging from language disability to severe autism. Mutation carriers typically display no dysmorphic features, but interestingly they can undergo regression at disease onset, characterised by a loss of initially acquired social and verbal milestones. NLG mutations thus clearly exemplify how regression does not necessarily imply the existence of environmental factors striking at the time when behavioural abnormalities become apparent, but simply stems from damage to neural networks becoming evident at the time when they should come “on-line” in order to support the harmonious growth and expansion of social cognition.

In summary, NLG mutation carriers display a variety of syndromes, ranging from X-linked MR without autism, to Asperger syndrome, autistic disorder of variable severity and PDD-NOS. Disease onset may be insidious or abrupt and regressive. These patients do not share any morphological or dysmorphic feature apt to spur a specific diagnostic investigation. The low frequency of these mutations and the lack of phenotypic traits suggesting the involvement of NLG3 and NLG4 do not encourage the clinical implementation of widespread genetic screens for these genes in patients with non-syndromic ASD.

### SH3 and multiple ankyrin repeat domains 3 gene (*SHANK3*)

The *SHANK3* gene is located on the telomeric terminal of chromosome 22q13.3 and encodes for a scaffolding protein found in the postsynaptic density (PSD) complex of excitatory synapses, where it binds directly to neuroligins. Two recent studies have shown a possible correlation between mutations or small cytogenetic rearrangements affecting *SHANK3* and an ASD phenotype mainly characterised by severe verbal and social deficits (table 2).^40^ ^41^ Similar to neuroligin mutations, SHANK3 mutations, deletions or duplications are rare, occurring in only 1.1% of patients with ASD. However, the genotype–phenotype correlation reported in both studies suggests that patients with autism with severe language and social impairment might be good candidates for *SHANK3* mutation screening. Five of seven cases reported to date carry deletions (142 kb to 4.36 Mb in size), of the terminal 22q13 region encompassing *SHANK*3, whereas the other two have a frameshift (E409X) and a missense mutation (Q321R).^40^ ^41^ Furthermore, rare non-synonymous variations present in the ASD group, but not in the control sample, were also found in both studies.^40^ ^41^ However, these variations were inherited from healthy parents and sometimes were also present in unaffected siblings, suggesting that they may confer vulnerability rather than play a dominant role in ASD pathogenesis. Indeed two of the mutations (R12C and R300C) were associated with severe language problems and social interaction difficulties. In vitro overexpression of these variants resulted in diminished colocalisation with the presynaptic marker protein Bassoon, suggesting nonsynaptic clustering of mutated SHANK3.^40^ Incomplete penetrance of these variants could explain their presence in other unaffected family members. Alternatively, they may cause the disease by acting synergistically with other susceptibility genes.

**Table 2:** *SHANK3* and autistic spectrum disorders

Reference	Patients with ASD, n	De novo mutations, n (%)	Mutations/deletions	Clinical phenotype
Durand *et al*^40^	227	3/227 (1.3)	142 kb del at 22q13, E409X, 800 kb del at 22q13*	Autism with severe language and social deficits
Moessner *et al*^41^	400	4/400 (1)	277 kb del at 22q13, 3.2 Mb del at 22q13,* 4.36 Mb del at 22q13, Q321R	Autism with non-verbal communication and social deficits
Total	627	7/627 (1.1)	—	—

ASD, autistic spectrum disorders.

*Parents have a balanced translocation.

It is interesting that both studies reported an inherited 22q13 deletion involving *SHANK3* (800 kb in Durand *et al*^40^ and 3.2 Mb at 22q13 in Moessner *et al*^41^), stemming from a paternal balanced translocation. In both studies, the probands with autism had siblings with partial 22q13 trisomy affected by disorders as different as Asperger syndrome with early language development^40^ and attention deficit hyperactivity disorder.^41^ These observations suggest that fine-tuning of *SHANK3* gene dosage may be crucial for the development of language and social cognition in humans.

### The neurexin 1 gene

Presynaptic neurexins induce postsynaptic differentiation in contacting dendrites, by interacting with their postsynaptic neuroligin partners.^9^ ^25^ The three neurexin genes (*NRXN1*, *NRXN2* and *NRXN3*, located at the human chromosome loci 2q32, 11q13 and 14q24.3-q31.1, respectively) have two independent promoters, which determine two mRNA classes: long mRNAs, encoding for α-neurexins and short mRNAs, encoding for β-neurexins.^42^ A mutational NRXN1β screening conducted by Feng *et al*^43^ on 264 patients with ASD identified two heterozygous missense mutations (S14L and T40S) and a GG insertion in position 26 of the corresponding protein (table 3). The two missense mutations were present in 4/264 (1.5%) patients with ASD and not in 729 controls, but they also occurred in first-degree relatives (parents and/or unaffected siblings), who displayed very heterogeneous phenotypes ranging from hyperactivity, depression and/or learning problems to apparently normal behaviour. Similarly, one of two chromosomal rearrangements affecting the *NRXN1* gene were also shown to be paternally inherited in one patient.^44^ The same study also reported two rare missense variants (L18Q and L748I) in 2 of 57 people with ASD.^44^ People with autism carrying the S14L mutations apparently have seizures and facial dysmorphisms.^43^ No clinical and family data were reported for patients carrying the insertion. In another study, a de novo heterozygous 300 kb deletion in the coding exons of the *NRXN1* gene was found in two affected sisters, who both received a diagnosis of autism.^45^ However, one girl was reported to be non-verbal, whereas the other had mild language regression.^45^ In a recent case–control study by Yan *et al*,^46^ five rare patient-specific variants were identified in 116 ASD individuals whereas only one additional variant (G28A) was also found in controls. Unfortunately, no clinical or family data are available for this study.

**Table 3:** Neurexin 1 and autistic spectrum disorders

Reference		Patients with ASD, n	De novo mutations, n (%)	Mutations/deletions		Clinical phenotype	
Feng *et al*^43^		264 (219M+45F)	0/192 (0)	S14L*, T40S,* 26insGG†	Autism with seizures and facial dysmorphism		
The Autism Genome Project Consortium^45^		196	2/196‡ (0.5)	300 kb del at 2p16	Autism with mild to severe spoken language deficits		
Kim *et al*^44^		57	0/57 (0)	ins(16;2)(q22.1;p16.1p16.3).* t(1;2)(q31.3;p16.3).§ L18Q,† L748I*	—		
Yan *et al*^46^		116	0/116 (0)	R8P, L13F, G28A,* c1024 +1 G→A,† T665I,† E715K†	—		
Total		633	1/633 (0.15)	—	—		

ASD, autistic spectrum disorders.

*Mutations found also in other non-affected family members or in controls.

†No clinical and pedigree information available; not specified if occurring de novo.

‡Counted as “1”, because affected members were consanguineous relatives from the same pedigree.

§Rearrangement localised ∼750 kb 5′ to the *NRXN1* locus.

The presence of deletions or other mutations also in unaffected siblings of patients with autism and even in controls,^43^ ^44^ ^46^ suggest that such mutations could confer vulnerability to ASD rather than causing the disease. In addition, the small number of studies performed to date on the putative link between neurexin genes and autism suggest caution in addressing potential genotype–phenotype correlations.

### The methyl-CpG-binding protein 2 gene

Methyl-CpG-binding protein 2 (MeCP2) is a transcriptional repressor that binds to methylated CpG dinucleotides generally located at gene promoters and recruits HDAC1 and other proteins involved in chromatin repression.^11^ De novo mutations of the *MECP2* gene located on chromosome Xq28 occur in 80% of female patients with Rett syndrome, a pervasive developmental disorder generally characterised by regression, autism, microcephaly, stereotyped behaviours, epilepsy and breathing problems, whereas in males, mutations are generally lethal.^10^ ^11^ Some mutations can also result in asymptomatic phenotypes, mild MR and verbal Rett variants.^11^ The clinical phenotype depends upon the type of mutation and the specific X-inactivation pattern, which is highly skewed in the presence of mutations affecting X-linked genes, such as *NLGN3* and *MECP2*, but is not significantly skewed in ASD families overall.^47^

Several groups have screened the *MECP2* gene for mutations in patients with non-syndromic ASD (table 4).Of 11 studies, 2 have identified de novo *MECP2* mutations in female patients with ASD: a de novo splice variant in intron 2 (IVS2 + 2delTAAG), a frameshift mutation (1157del41) and a nonsense mutation (R294X).^48^ ^49^ Interestingly, the phenotype associated with this mutation includes MR but not epilepsy, microcephaly, stereotypies or any of the symptoms characteristic of Rett syndrome; regression was present in one patient, but not in the other two.^48^ ^49^ Another study identified two de novo mutations (R133C and R453X) in girls with ASD fulfilling the criteria for the preserved speech variant of Rett syndrome.^50^ The remaining eight studies were either entirely negative or reported missense, intronic or 3′-untranslated region variants potentially of functional interest; either these were reported to be inherited from one of the parents or it was not specified whether they were inherited or de novo.^51^^–^58 Overall, *MECP2* mutations are rare in the autistic population, accounting for 0.8–1.3% of cases in the female ASD population (ie, 3/378 or 5/397, respectively, depending whether patients from the Zappella *et al* study^50^ are included (table 4) and not including subjects from the Shibayama *et al*^53^ study for which no male:female ratio is provided). Importantly, female patients with ASD carrying *MECP2* mutations appear mentally retarded, but do not display any clinical trait resembling Rett syndrome. Signs and symptoms more typical of Rett syndrome may be of later onset, requiring that female patients with ASD be clinically monitored over time.^59^

**Table 4:** *MECP2* and autistic spectrum disorders

Reference	Patients with ASD, n	De novo mutations, n (%)	Mutations/deletions	Clinical phenotype
Lam *et al*^48^	21 (all F)	1/21 (4.8)	IVS2+2delTAAG	Autism and MR. No regression, epilepsy or microcephaly
Vourc’h *et al*^51^	59 (42M,17F)	—	—	—
Beyer *et al*^52^	202 (154M,48F)	—	—	—
Carney *et al*^49^	69 (all F)	2/69 (2.9)	1157del41, R294X	Autism, MR and history of regression. No stereotypies, epilepsy or microcephaly
Zappella *et al*^50^	19 (all F)	2/19 (4.7)	R133C, R453X	Preserved speech variant of Rett syndrome
Shibayama *et al*^53^	24‡	0/24 (0)	c1638 G→C,* c 6809 T→C,* P376R†	—
Lobo-Menendez *et al*^54^	99 (58M,41F)	–	–	—
Li *et al*^55^	65 (49M,16F)	—	—	—
Xi *et al*^56^	31 (all M)	0/31 (0)	1 3′UTR variant*	—
Harvey *et al*^57^	401 (266M,135F)	—	—	—
Coutinho *et al*^58^	172 (141M,31F)	0/172 (0)	12 3′UTR variants.† 2 intronic variants,† G206A*	—
Total	397F, 741M‡	5/397 females (1.3), 0/741 males	—	—

ASD, autistic spectrum disorders; MR, mental retardation; UTR, untranslated region.

*Mutations found also in other non-affected family members or in controls.

†Not specified if occurring de novo; no pedigree information available.

‡The study by Shibayama *et al*^53^ did not provide an M:F ratio and was excluded from the total.

### The homeobox A1 gene

*HOXA1* is a homeobox gene located at human chromosome locus 7p15.3, essential to the development of head and neck structures, including hindbrain, ear, and occipital and hyoid bones.^60^ ^61^ Homozygous recessive stop-codon mutations of this gene have been found in nine people belonging to four consanguineous families from Saudi Arabia and one from Turkey.^62^ ^63^ Two different mutations were identified: a 84C→G mutation results in the introduction of a stop codon in the Turkish person, and a 175–176insG causes a reading frame shift and premature protein truncation in the Saudi Arabian patients. The probands of these families showed a similar phenotype characterised by horizontal gaze abnormalities, deafness, focal weakness, hypoventilation, vascular malformations of the internal carotid arteries and cardiac outflow tract, MR and autism in some, but not all patients: this clinical set of symptoms and malformations was named Bosley–Salih–Alorainy syndrome (BSAS).^63^

### The phosphatase and tensin homologue gene

*PTEN* is a tumour suppressor gene located on human chromosome 10q23, influencing G1 cell-cycle arrest and apoptosis. In the central nervous system, *PTEN* inactivation results in excessive dendritic and axonal growth with increased numbers of synapses.^64^ Germline mutations resulting in *PTEN* haploinsufficiency thus facilitate cell-cycle progression and oncogenesis, leading to macrocephaly/macrosomy and to cancer development, respectively.^65^ Germline *PTEN* mutations have been found in approximately 80% of individuals diagnosed with Cowden syndrome, accompanied by enhanced risk for breast, endometrial and thyroid malignancies.^65^ People with other related hamartoma disorders, such as Bannayan–Riley–Ruvalcaba syndrome, Proteus and Proteus-like syndromes display germline *PTEN* mutations in 60%, 20% and 50% of cases, respectively.^65^ In addition, somatic *PTEN* mutations have been found in a variety of human tumours.^65^ Interestingly, genetic syndromes due to *PTEN* germline haploinsufficiency, in addition to excessive, dysplastic or neoplastic growth, are often characterised by autism or MR and progressive macrocephaly, as initially reported by Goffin *et al*.^66^ A study by Butler *et al*^67^ identified three de novo heterozygous *PTEN* germline mutations in 18 (16.6%) people with macrocephaly and autism (table 5). The three newly identified mutations all lead to missense changes in evolutionarily conserved aminoacid residues. Others found *PTEN* mutations in different percentages of macrocephalic patients with ASD^68^^–^70 (in one case also displaying polydactyly of both feet).^70^

**Table 5:** *PTEN* and autistic spectrum disorders

Reference	Patients with ASD, n (%)	De novo mutations, n	Mutations/deletions	Clinical phenotype
Goffin *et al*^66^	1 (M)	0	Y178X*	Cowden syndrome with autism and progressive macrocephaly
Butler *et al*^67^	18 (13M,5F) all macrocephalic	3/18 (16.6)	H93R, D252G, F241S	Extreme macrocephaly and macrosomy
Herman *et al*^6869^	71 (57M,14F), including 19 macrocephalic	2/71 (2.8); 2/19 macrocephalic (10.5)	530 insT; R130X	Macrocephaly, autism and developmental delay
Buxbaum *et al*^70^	88 (76M,12F) all macrocephalic	1/88 (1.1)	D326N	Macrocephaly (+9.6 SD), polydactyly at both feet, autism, MR, language delay
Total	126 macrocephalic	6/126 (4.7) macrocephalic patients with ASD		

ASD, autistic spectrum disorders; MR, mental retardation.

*Mutation found also in the patient’s mother (Cowden syndrome).

Cases of ASD with *PTEN* mutations are invariably characterised by severe to extreme macrocephaly (ie, cranial circumference >97th percentile or +2 SD, but *PTEN* mutation carriers with ASD typically display ⩾+3 SD). Macrocephaly has been consistently found in approximately 20% of patients with autism recruited in independent samples.^71^^–^75 Head circumference in these patients with ASD is typically normal at birth and an overgrowth seemingly develops over the first few years of life.^76^ Interestingly, the majority of macrocephalic patients are actually macrosomic, as head size is significantly correlated with excessive height and weight in this subgroup of patients with autism.^77^ The incidence of *PTEN* de novo mutations in these macrocephalic/macrosomic patients with ASD can be estimated at 4.7% (6/126) (table 5). Importantly, these patients are at increased risk of developing a variety of *PTEN*-related cancers during adulthood.

## GUIDELINES FOR GENETIC COUNSELLING

Based on the information summarised above, we have defined a set of practical guidelines that clinical geneticists should consider, together with recently reviewed ethical issues,^78^ when providing genetic counselling to parents of autistic children.

- If a couple has a non-syndromic autistic child, the probability that a second child will also be autistic is approximately 5–6%.
- A karyotype and fragile-X testing must be requested for all patients with ASD.
- Clinicians should be aware that prenatal genetic testing typically raises unrealistic expectations in parents of children with autism because, with the exception of rare gross chromosomal rearrangements, the genetic or genomic anomalies responsible for ASD are not detectable using current prenatal screening methods.
- In the presence of dysmorphic features and evident neurological symptoms, it is reasonable to suspect chromosomal rearrangements even if the karyotype appears normal. Depending upon availability and cost, a BAC or oligo array-based CGH analysis is strongly advised in these cases. As these technologies will become progressively more available, it will be important not to restrict them to patients with dysmorphia, as microdeletions and microduplications are also common among patients with idiopathic, non-dysmorphic ASD.
- Patients with *NLG3* and *NLG4* mutations do not share any specific clinical features and the frequency of these mutations is sufficiently low that widespread screening for neuroligin mutations do not seem to be clinically justified.
- For the same reasons and owing to the small number of studies performed to date, widespread screening for the neuroxin genes are also not advisable at present.
- Genetic screening programmes for the *SHANK3* gene can potentially be implemented for patients with severe verbal and social deficits, bearing in mind that some *SHANK3* mutations have also been found to be transmitted to unaffected siblings of ASD probands (see table 1 in Moessner *et al*^41^).
- *MECP2* genetic testing should be performed in all girls with ASD and MR, remembering that clinicians should not expect these patients to bear any resemblance to Rett syndrome (ie, girls with autism carrying *MECP2* mutations will have normal head size, a history of regression may be present or absent, and they will usually show no stereotypies or seizures).
- *HOXA1* mutations are recessive. It is thus reasonable to screen only patients from consanguineous families, when presenting with autism associated with deafness, troncoencephalic anomalies and especially horizontal gaze abnormalities.
- The *PTEN* gene should be screened for mutations in all children with autism and extreme macrocephaly (⩾3 SD). Because of the tumour susceptibility produced by *PTEN* haploinsufficiency, it is strongly advised to start an oncological monitoring programme immediately for patients with ASD carrying *PTEN* mutations.
- If the parents of a macrosomic autistic child (carrying no genomic rearrangement or mutation affecting the genes discussed above) decide to have another child, clinicians should monitor weight, height and head circumference of the baby sibling at regular intervals from birth to 2 years of age (ie, during the clinical “latency” phase that precedes the onset of autistic symptoms). If the neonate displays a progressive overgrowth of head and body size compared with age-specific and sex-specific population norms, it is advisable to start an early intervention programme, wherever available, with the aim of stimulating social cognition at 18–24 months of life.

The design of behavioural intervention programmes targeted to address ASD signs and symptoms as early as possible (ie, before the age of 3 years) is being actively pursued in many clinical centres, in order to intervene during the critical period of maximum plasticity in postnatal brain development.^79^ Within this context, working in conjunction with paediatricians and child neuropsychiatrists, clinical geneticists can significantly contribute not only to the diagnostic process, but also to successful implementation of the most appropriate behavioural intervention programme. By designing and implementing clinically targeted genetic screens, as proposed in our guidelines, clinical geneticists will provide medically relevant information while decreasing the level of anxiety and guilt in the parents, thereby increasing their compliance with the pharmacological and behavioural treatment of their child with autism. Transferring the latest knowledge from autism genetic research into clinical practice may not yet provide all the definitive answers that investigators and clinicians would have wished to have reached at this time, but can certainly contribute to raise the clinical management of these patients and their families beyond current standards.
